# 4446 Association between treatment of asymptomatic Trichomonas vaginalis infection and preterm delivery

**DOI:** 10.1017/cts.2020.108

**Published:** 2020-07-29

**Authors:** Anna Marie Pacheco Young, Gayane Yenokyan, Jenell Coleman

**Affiliations:** 1 Johns Hopkins University School of Medicine; 2 Johns Hopkins Bloomberg School of Public Health; 3 Johns Hopkins Department of OBGYN

## Abstract

OBJECTIVES/GOALS: *Trichomonas vaginalis* (TV) has a prevalence of 26% in Baltimore and is associated with preterm delivery (PTD). Yet screening and treatment of TV is not advised due to conflicting data on harms. Our goal is to investigate the association between asymptomatic TV treatment and PTD. METHODS/STUDY POPULATION: This is a retrospective cohort study of women who delivered a child at The Johns Hopkins Hospital between 7/1/16 – 11/19/19. Exclusion criteria included multiple gestation, stillborn, miscarriage, diabetes, hypertension/ preeclampsia, HIV, and history of PTD. Chart review and ICD-10 diagnosis codes were used to collect data on demographics, STI test results, lab encounter diagnoses, STI treatment during pregnancy, and labor encounter diagnoses. Preliminary analysis for crude incidence of PTD in asymptomatic and symptomatic women treated for TV was performed using TriNetx, a global research network compiling all de-identified data within the Hopkins system. RESULTS/ANTICIPATED RESULTS: Three hundred and eighty women were tested for TV, 240 (63%) were asymptomatic and 140 (37%) women were symptomatic. Mean ages were 26 (SD:5) and 26 (SD:5) years, respectively. Black women comprised 87% of the asymptomatic cohort and 93% of the symptomatic cohort. Women of Hispanic ethnicity were represented by 4% of the asymptomatic cohort and 7% of the symptomatic cohort. Crude incidence of PTD was 4.1% among asymptomatic women and 7.1% among symptomatic women. Incidence ratio comparing asymptomatic PTD incidence to symptomatic PTD incidence was 0.58 with 95% CI (0.22, 1.56). DISCUSSION/SIGNIFICANCE OF IMPACT: Preliminary data from our study suggests there is no difference in PTD between asymptomatic and symptomatic women treated for TV. Future steps include multiple linear regression using a larger dataset. These preliminary data suggest TV should be considered for screening during pregnancy.

